# Improving the production of 22-hydroxy-23,24-bisnorchol-4-ene-3-one from sterols in *Mycobacterium neoaurum* by increasing cell permeability and modifying multiple genes

**DOI:** 10.1186/s12934-017-0705-x

**Published:** 2017-05-22

**Authors:** Liang-Bin Xiong, Hao-Hao Liu, Li-Qin Xu, Wan-Ju Sun, Feng-Qing Wang, Dong-Zhi Wei

**Affiliations:** State Key Laboratory of Bioreactor Engineering, Newworld Institute of Biotechnology, East China University of Science and Technology, Shanghai, 200237 China

**Keywords:** *Mycobacterium*, 22-Hydroxy-23,24-bisnorchol-4-ene-3-one (4-HBC), *mmpL3*, *choM*, *cyp125*, *fadA5*, Sterol catabolism

## Abstract

**Background:**

The strategy of modifying the sterol catabolism pathway in mycobacteria has been adopted to produce steroidal pharmaceutical intermediates, such as 22-hydroxy-23,24-bisnorchol-4-ene-3-one (4-HBC), which is used to synthesize various steroids in the industry. However, the productivity is not desirable due to some inherent problems, including the unsatisfactory uptake rate and the low metabolic efficiency of sterols. The compact cell envelope of mycobacteria is a main barrier for the uptake of sterols. In this study, a combined strategy of improving the cell envelope permeability as well as the intracellular sterol metabolism efficiency was investigated to increase the productivity of 4-HBC.

**Results:**

*MmpL3*, encoding a transmembrane transporter of trehalose monomycolate, is an important gene influencing the assembly of mycobacterial cell envelope. The disruption of *mmpL3* in *Mycobacterium neoaurum* ATCC 25795 significantly enhanced the cell permeability by 23.4% and the consumption capacity of sterols by 15.6%. Therefore, the inactivation of *mmpL3* was performed in a 4-HBC-producing strain derived from the wild type *M. neoaurum* and the 4-HBC production in the engineered strain was increased by 24.7%. Subsequently, to enhance the metabolic efficiency of sterols, four key genes, *choM1*, *choM2*, *cyp125*, and *fadA5*, involved in the sterol conversion pathway were individually overexpressed in the engineered *mmpL3*-deficient strain. The production of 4-HBC displayed the increases of 18.5, 8.9, 14.5, and 12.1%, respectively. Then, the more efficient genes (*choM1*, *cyp125*, and *fadA5*) were co-overexpressed in the engineered *mmpL3*-deficient strain, and the productivity of 4-HBC was ultimately increased by 20.3% (0.0633 g/L/h, 7.59 g/L 4-HBC from 20 g/L phytosterol) compared with its original productivity (0.0526 g/L/h, 6.31 g/L 4-HBC from 20 g/L phytosterol) in an industrial resting cell bio-transformation system.

**Conclusions:**

Increasing cell permeability combined with the co-overexpression of the key genes (*cyp125*, *choM1*, and *fadA5*) involved in the conversion pathway of sterol to 4-HBC was effective to enhance the productivity of 4-HBC. The strategy might also be useful for the conversion of sterol to other steroidal intermediates by mycobacteria.

**Electronic supplementary material:**

The online version of this article (doi:10.1186/s12934-017-0705-x) contains supplementary material, which is available to authorized users.

## Background

Mycolata actinomycetes, including *mycobacteria* [1], rhodococci [2, 3] and *gordonia* [4], are mycolic acids-rich bacteria and can catabolize natural sterols as carbon and energy sources [5]. The interruptions in the sterol metabolism pathway of mycobacteria lead to the accumulation of some important intermediates which can be used as ideal precursors to synthesize valuable steroidal pharmaceuticals [6]. For example, the C_22_ steroids, including 22-hydroxy-23,24-bisnorchol-4-ene-3-one (4-HBC), 22-hydroxy-23,24-bisnorchol-1,4-dien-3-one (1,4-HBC) and 9,22-dihydroxy-23,24-bisnorchol-4-ene-3-one (9-OHHBC), are suitable precursors for the synthesis of progestational and adrenocortical hormones [7–9].

Sterols are a kind of hydrophobic lipid compounds. In the nature, the uptake of sterols by mycobacteria mainly relies on the direct contact between the particles of sterol and the cell envelope [10]. As one of the distinctive structure of mycobacteria, the complex structure of mycolyl-arabinogalactan-peptidoglycan in the cell envelope core forms an asymmetrical and non-fluid layer outside of the cell membrane [11–14]. Moreover, the surface of this structure is decorated with a variety of non-covalently associated capsular lipids, including trehalose monomycolate (TMM), trehalose dimycolate (TDM) as well as a capsule-like coat of polysaccharide and protein [12, 14]. The polar glycolipids layer on the cell envelope surface of mycobacteria provides an optimum surface contact for material exchange [15]. However, the major frame of the complex structure in the cell envelope core guarantees a low permeability of the cells and is a main negative factor for the uptake of sterol into mycobacteria [16]. In the presence of synthesis inhibitors of the cell envelope, such as vancomycin and glycine, the uptake of sterol through the cell envelope shows significant improvements [17–20]. As a result, the sterol utilization rate and the productivity of target steroidal intermediates are remarkably increased in mycobacterial cells. However, these strategies can hardly be applied due to the high cost of the added inhibitors in large-scale production.

Mycolic acids, which accounted for 40–60% of cell dry weight, are known to play an essential role in the formation of the cell envelope surface and the cell envelope core [21]. Through a unique biosynthesis pathway in the mycobacterial cytoplasm, synthesized mycolic acids are conjugated with trehalose to form TMM, which acts as a primary mediator for adjusting the hydrophobicity of the cell envelope [14]. Then, these TMM molecules are transported from the cytoplasmic production site into the periplasm. Finally, they are transferred to the cell envelope region for the assembly of mycolic acid-related structures and other molecules, including glucose monomycolate, glycerol monomycolate, the above mentioned TDM and the critical mycolyl-arabinogalactan-peptidoglycan complex. The transmembrane transport of TMM requires some transport proteins [14]. The details of the transport process were still unclear [22], but it was revealed that the MmpL3 was probably implicated in the transport of the essential TMM from cytoplasm to periplasm in *Mycobacterium tuberculosis* [14, 21]. Interferences with the MmpL3 function in mycobacterial cells were expected to inhibit the TMM translocation to the cell envelope, thus leading to an improvement in the cell permeability. The uptake of sterols as well as the productivity of target steroidal intermediates is possibly increased accordingly. In addition, through the augmentation of key genes in engineered *M. neoaurum* strains, the production of androst-4-ene-3,17-dione (AD), androst-1,4-diene-3,17-dione (ADD) and 9α-hydroxy-4-androstene-3,17-dione (9-OHAD) was remarkably increased [23, 24]. The metabolic engineering strategy of overexpressing genes directly involved in the conversion pathway has proved to be an applicable way to increase the production of target metabolites.

In this study, the changes of cell permeability and sterol consumption after deleting of *mmpL3* in *M. neoaurum* ATCC 25795 were determined. Meanwhile, the improvement in 4-HBC production caused by the deficiency of *mmpL3* in its derived 4-HBC-producing strain was explored. Additionally, to further enhance the productivity of 4-HBC, we evaluated the effect of the individual overexpression of some key genes including *cyp125* [25, 26], *choM1*, *choM2* [23] and *fadA5* [27] in the conversion pathway of sterols on the 4-HBC production. Then, the influence of their combinatory overexpression on the 4-HBC production was assessed in an industrial resting cell bio-transformation system.

## Results

### Comparison of the *mmpL3* genome region in *mycobacteria*

The inhibition of MmpL3, a membrane transporter involved in the transmembrane transport of trehalose monomycolate (TMM) from cytoplasm to periplasm resulted in the accumulation of TMM in cytoplasm of *M. tuberculosis* H37Rv [14]. This modification would interfere with the normal assembly of the cell envelope [14] and possibly cause an improvement in cell envelope permeability, thus leading to a corresponding increase in the uptake of sterols by mycobacteria [17–20]. Therefore, we firstly located the possible *mmpL3* region in the genome of *M. neoaurum* ATCC 25795, which is a highly homologous strain of well-known steroidal intermediate producer strains [28, 29]. Then, the possible *mmpL3* genome region in *M. neoaurum* ATCC 25795 was compared with the homologous region in *Mycobacterium neoaurum* NRRL B-3805, *Mycobacterium neoaurum* VKM Ac-1815D, and *M. tuberculosis* H37Rv. The parameter of sequence identity was used to preliminarily assess the functional homology between the *mmpL3* in *M. neoaurum* and its homologous genes.

**Fig. 1:**
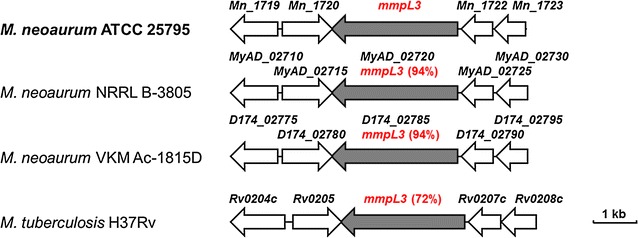
Localization of the *mmpL3* homologues in the genome of *M. neoaurum* ATCC 25795 and other mycobacteria. The size and direction of genes from the predicted genome information were displayed as an arrow according to the scale. The percentages, such as 94 and 72%, indicate the sequence identity of *mmpL3* from *M. neoaurum* ATCC 25795 with the homologs in *M. neoaurum* NRRL B-3805, *M. neoaurum* VKM Ac-1815D, and *M*. *tuberculosis* H37Rv

A transmembrane transport protein encoded by *Mn_1721* (GeneBank: NZ_JMDW01000016.1; Region: 7540…4670, 2871-bp) in *M. neoaurum* ATCC 25795 can be annotated as a homologous protein with the MmpL3, which has been identified in the other two mycobacteria [14, 21]. The gene shows a high nucleotide sequence identity with *MyAD*_*02720* (2871-bp, 94%, GeneBank ID: AMO04272.1) from *M. neoaurum* NRRL B-3805, *D174_02785* (2871-bp, 94%, GeneBank ID: AHC23578.1) from *M. neoaurum* VKM Ac-1815D, and *mmpL3* (*Rv_0206*, 2835-bp, 72%, GeneBank ID: NP_214720.1) from *M. tuberculosis* H37Rv (Fig. 1; Additional file 1: Table S1). Moreover, the genomic location of *mmpL3* is highly conserved among the listed *mycobacteria*. The *mmpL3* from *M. neoaurum* ATCC 25795 is located between *Mn_1720* and *Mn_1722*, which also show the high identity (70–93%) and similar organizations with corresponding genes from the other three strains in the genus *Mycobacterium*. Comparison results of the *mmpL3* genome region suggest that the function of MmpL3 is possibly conserved in mycobacteria. Like the disruption of its homologous genes in *M. tuberculosis* H37Rv, the deletion of *mmpL3* in *M. neoaurum* ATCC 25795 is probably beneficial to the improvement in cell envelope permeability.

### Deletion of *mmpL3* affects cell envelope permeability and cholesterol utilization

**Fig. 2:**
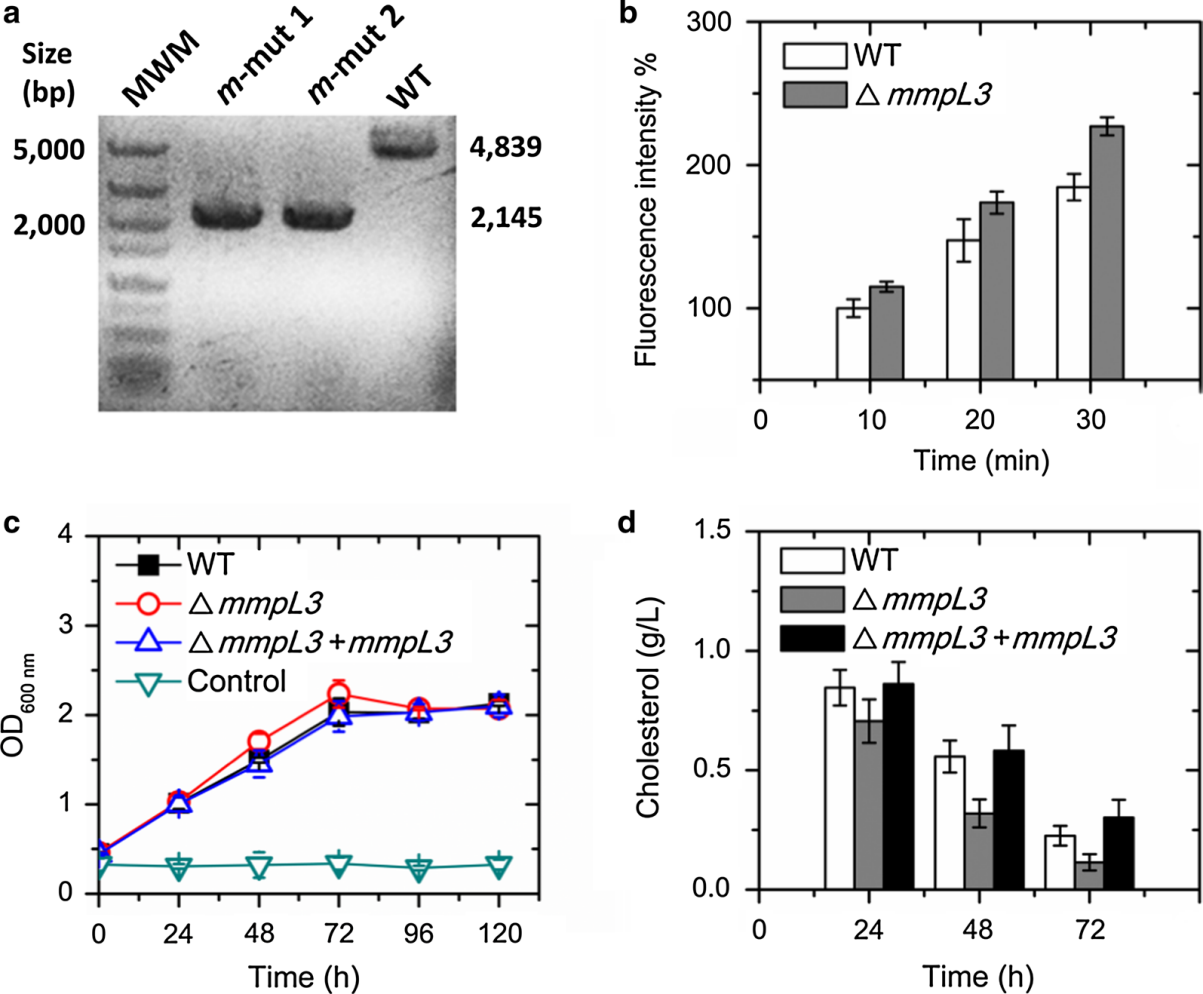
Effects of deleting *mmpL3* on cell permeability and the cholesterol utilization. **a** Evidence for allelic replacement at the *mmpL3* locus of *M. neoaurum* ATCC 25795. The wild-type (WT) 4839-bp sequence was replaced by a 2145-bp fragment ligated with a 1074-bp upstream sequence and a 1071-bp downstream of the *mmpL3* (*m*) gene, thus resulting in the *mmpL3*-deficient *M. neoaurum* (*m*-mut1 and *m*-mut2). *MWM* molecular weight marker. **b** Effects of MmpL3 disruption on cell permeability. Diluted cell suspensions were stained with fluorescein diacetate (FDA) and then the mixtures were detected by a fluorescence spectrophotometer. **c** Growth characteristics of the wild-type *M. neoaurum* ATCC 25795 (WT, *squares*), the deficiency strain of *mmpL3* in the WT (Δ*mmpL3*, *open circles*) and the complementation strain of *mmpL3* in the Δ*mmpL3* (Δ*mmpL3*+*mmpL3*, *triangles*) cultured in MM with 1.0 g/L cholesterol. The control is the medium containing 1.0 g/L cholesterol without inoculum. **d** Quantitative determination of residual cholesterol from the three strains cultured in MM with 1.0 g/L cholesterol. Data represent mean ± standard deviation of three measurements

**Fig. 3:**
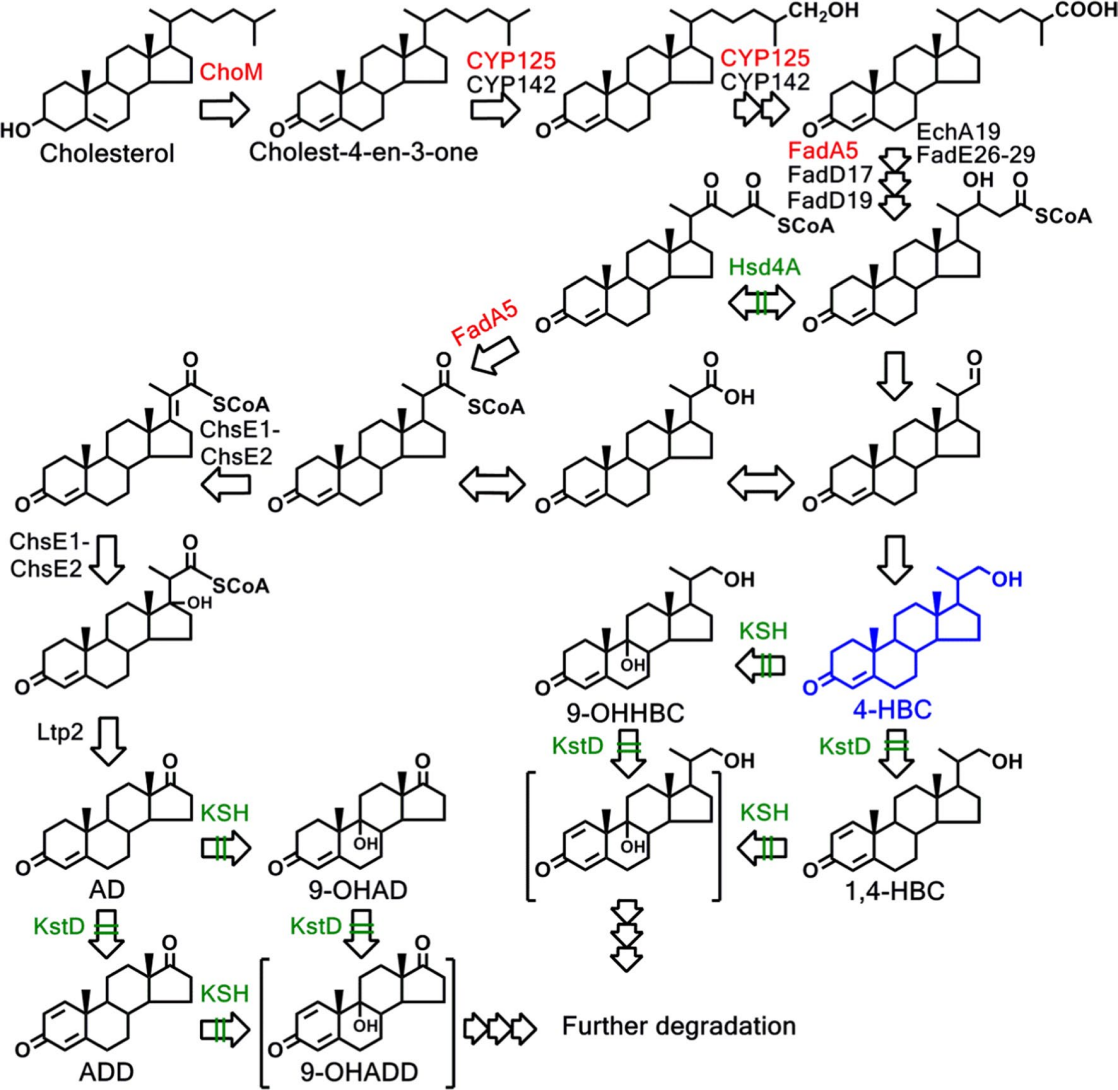
Schematic profiles of the conversion pathway of sterol to 4-HBC. Sterols share a common and conserved degradation pathway. Here, the cholesterol was used as the model substrate of the sterol catabolism pathway. The disruptions of several enzymes to block the sterol catabolism pathway, resulting in the accumulation of 4-HBC, was colored with *green font*. The genes colored with *red font* in the upstream conversion pathway of sterol to 4-HBC were individually overexpressed in the strain WIIIΔ*mmpL3*

In order to assess the effect of MmpL3 on cell envelope permeability, the *mmpL3* was deleted from the genome of wild-type strain *M. neoaurum* by allelic replacement (Fig. 2a). The fluorescence intensity of the Δ*mmpL3* strain labeled by fluorescein diacetate (FDA) in minimal medium (MM) was analyzed. Cell permeability of the *mmpL3*-deleted strain showed an improvement of 23.4% at 30 min compared with that of the wild-type strain (Fig. 2b). Meanwhile, the growth of the strain Δ*mmpL3* did not decline significantly, but displayed an abnormal rise compared with that of the wild-type strain was observed (Fig. 2c). The slight acceleration in the growth rate of the strain Δ*mmpL3* was inconsistent with the similar disruption study in *M. smegmatis* [21] as well as our previous phenotype analysis of the gene deletion strains of *M. neoaurum* [23, 30]. This might be interpreted as follows. The improved cell permeability increased the supplement of steroids after the deletion of *mmpL3*. To confirm this speculation, cholest-4-en-3-one was selected as a label to evaluate the steroids uptake because it was the first metabolite in the oxidation of cholesterol oxidases (Fig. 3) and could be detected by common UV detector, but the cholesterol could not be detected by common UV detector. The result indicated that the uptake of cholest-4-en-one was significantly enhanced by 33.7% after the deletion of *mmpL3* (Additional file 2: Figure S1a, 1b). This result was consistent with the enhanced cell permeability characterized by FDA. Besides, we further analyzed the cholesterol utilization of the wild-type strain *M. neoaurum*, the strain Δ*mmpL3* and the complemented strain Δ*mmpL3*+*mmpL3* (Fig. 2d).

Comparison results showed that the utilization of cholesterol in the strain Δ*mmpL3* was higher than that of the strain Δ*mmpL3*+*mmpL3* and the wild-type strain. The residual cholesterol concentration in the medium of strain Δ*mmpL3* was about 0.71 g/L at 24 h, 0.32 g/L at 48 h, and 0.11 g/L at 72 h, while the concentrations of cholesterol in the medium of the wild-type stain and the strain Δ*mmpL3*+*mmpL3* were 0.85–0.86, 0.56–0.58 and 0.23–0.30 g/L, respectively. The cholesterol utilization at 24, 48, and 72 h respectively showed the improvements of 93.3, 54.5, and 15.6%. In addition, the deletion of *mmpL3* caused no obvious inhibition on cell growth of the strain (Fig. 2c). In a word, the deficiency of *mmpL3* in the wild-type *M. neoaurum* showed an improvement in cell permeability and the cholesterol utilization.

### Improvement in the 4-HBC productivity in the engineered strain by deletion of *mmpL3*

To further evaluate whether the deletion of *mmpL3* could increase the productivity of the target intermediate 4-HBC, the *mmpL3* was deleted in a typical 4-HBC-producing strain WIII (Δ*kshA*Δ*hsd4A*Δ*kstD123*) [8] to generate a strain WIIIΔ*mmpL3*.

**Fig. 4:**
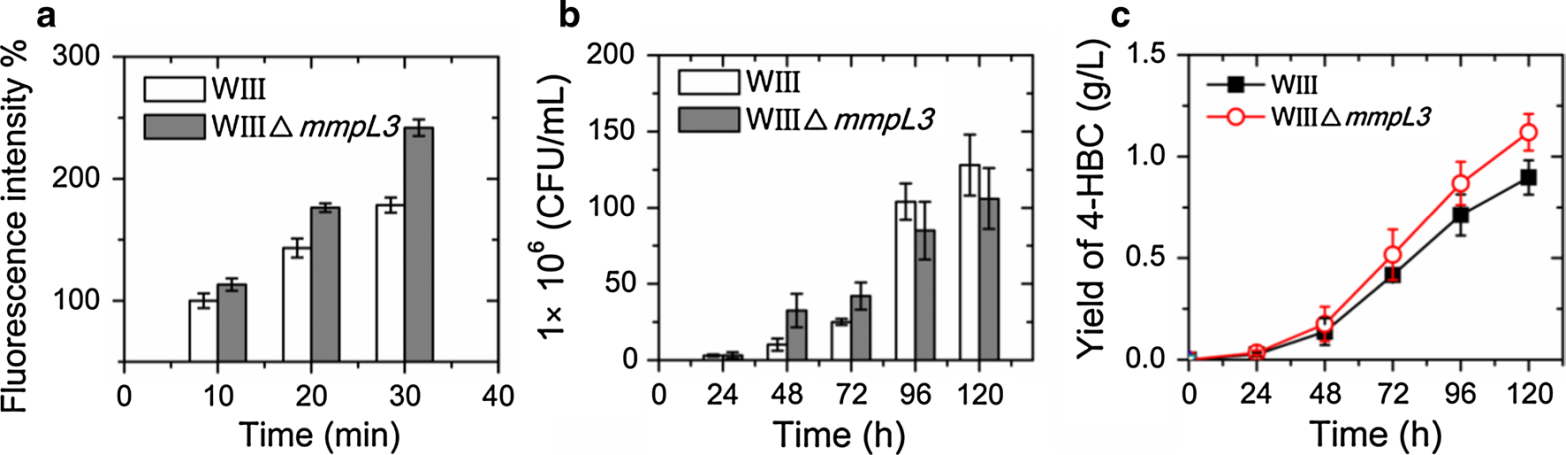
Effects of deleting *mmpL3* on the improvement in 4-HBC production in the engineered mycobacterial strains. **a** Improvement in cell permeability in the *mmpL3*-deleted strain. The engineered strains were stained with FDA, incubated at 32 °C for 10 min and then the mixtures were analyzed by the fluorescence spectrophotometer. **b** Growth of the previously constructed 4-HBC-producing strain (WIII) and the *mmpL3*-deleted strain WIIIΔ*mmpL3* in MYC/02 medium with 2.0 g/L phytosterol. *CFU* colony forming unit. **c** Quantitative analyses of the 4-HBC production of the aerobic bioconversion by 4-HBC-producing strains in MYC/02 medium with 2.0 g/L phytosterol. Data represent the mean ± standard deviation of three measurements

In order to determine whether the deletion of *mmpL3* could improve cell permeability of the 4-HBC-producing strain WIII, the fluorescence intensity in cells WIII and WIIIΔ*mmpL3* in MYC/02 medium was analyzed. We found that the cell permeability of the strain WIIIΔ*mmpL3* was improved significantly compared to that of the strain WIII (Fig. 4a). The increase in the fluorescence intensity of the strain WIIIΔ*mmpL3* compared to that of the strain WIII ranged from 13.2 to 35.5% when incubation time was increased from 10 to 30 min, indicating that the cell envelope assembly in the *mmpL3*-deleted cells might be damaged to some degree.

Moreover, the growth of strain WIIIΔ*mmpL3* showed some reduction after 96 h (Fig. 4b), but the production of target intermediate 4-HBC in the *mmpL3*-deleted strain showed an improvement. The deletion of this gene increased the production of 4-HBC by 24.7% from 0.90 to 1.12 g/L at 120 h (Fig. 4c). In addition, the consumption rate of phytosterol substrates in the strain WIIIΔ*mmpL3* also showed an obvious improvement after 96 h of biotransformation (Additional file 2: Figure S2). These results confirmed that the deletion of *mmpL3* did cause an improvement in cell permeability, thus leading to an increase of 4-HBC production in the engineered *mmpL3*-deficient strain.

### Enhancement of 4-HBC productivity by overexpressing key genes involved in the sterol catabolic pathway of the engineered strain

We significantly increased the conversion of phytosterols to AD and ADD by overexpression of cholesterol oxidases (ChoM1 and ChoM2) catalyzing the initial oxidation of sterol to sterone [23]. To further improve the production of the target intermediate 4-HBC, four genes, including *choM1*, *choM2*, a *cyp125* gene encoding a cytochrome P450 enzyme [25, 26] and a *fadA5* gene encoding a thiolase [27], in the conversion process of sterol to 4-HBC, were individually overexpressed in the engineered strain WIIIΔ*mmpL3* (Fig. 3).

**Fig. 5:**
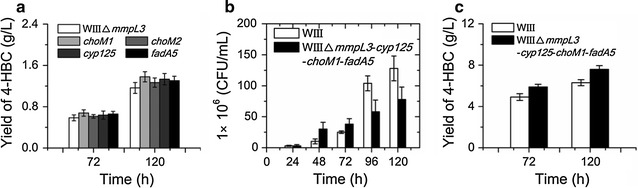
Enhancement of the 4-HBC productivity by overexpressing the genes in the sterol conversion pathway. **a** Assessment of the 4-HBC production for overexpressing the genes in the upstream conversion pathway of sterol to 4-HBC. **b** Growth of the WIIIΔ*mmpL3*-*cyp125*-*choM1*-*fadA5* (co-overexpression of *cyp125*, *choM1*, and *fadA5* in the strain WIIIΔ*mmpL3*) and its ancestral strain WIII in MYC/02 medium with 2.0 g/L phytosterol. **c** Assessment of the 4-HBC production in the constructed 4-HBC-producing strains. A “cyclodextrin-resting cell” system with 20 g/L of phytosterol was used to determine the productivity of the engineered strains. Data represent the mean ± standard deviation of three measurements

As shown in Fig. 5, the cells overexpressing one of these genes showed higher 4-HBC productivities than their parental strain WIIIΔ*mmpL3* after 120 h of biotransformation (Fig. 5a). Among these genes, overexpressing of *choM1* showed the greatest enhancement of 4-HBC (18.5%), followed by the overexpression of *cyp125* (14.5%) and *fadA5* (12.1%). Overexpression of *choM2* showed the lowest improvement (8.9%) effect in the 4-HBC production. Therefore, the more efficient genes *cyp125*, *choM1* and *fadA5* were co-overexpressed to further strengthen the efficiency of the sterol catabolism in the strain WIIIΔ*mmpL3*. The growth of the engineered strain WIIIΔ*mmpL3*-*cyp125*-*choM1*-*fadA5* (co-overexpression of *cyp125*, *choM1* and *fadA5* in the strain WIIIΔ*mmpL3*) showed a significant decline compared with that of its ancestral strain WIII after 96 h (Fig. 5b). In order to confirm the effect of co-overexpression of the three key genes accurately, a “cyclodextrin-resting cell” system which had been applied in the industry [31] was employed here to evaluate the productivity of 4-HBC in the constructed strain. The strain WIIIΔ*mmpL3*-*cyp125*-*choM1*-*fadA5* yielded 7.59 g/L 4-HBC with the productivity of 0.0633 g/L/h after 120 h transformation, whereas the strain WIII yielded 6.31 g/L 4-HBC with the productivity of 0.0526 g/L/h (Fig. 5c). The combined modifications significantly improved the productivity of the target intermediate 4-HBC by 20.3% in the industrial resting cell bio-transformation system.

## Discussion

The diversity of metabolism in microorganisms, animals and plants provides us a huge database with enormous valuable natural products and metabolic intermediates [32]. Some natural products in organisms can be obtained directly using suitable extraction methods, but the concentrations of most of the metabolic intermediates are always low in organisms [33, 34].

As a traditional strategy to increase the production of target metabolites, metabolic engineering methods were used to increase the metabolism efficiency of the substrate to target metabolites [23, 24, 30]. Overexpression of key genes in the sterol catabolic pathway is effective to improve the productivity of the target 4-HBC. However, the strategy requires an expression vector of pMV261, which is only suitable for the expression of a few genes [30]. Meanwhile, the expression plasmids in the cells may interfere with the subsequent modifications of the engineered strains. Notably, most of the key genes involved in the sterol uptake and metabolism are mainly distributed in a highly conserved gene cluster and the genes in the cluster exists as operons [35]. Therefore, in order to avoid the usage of expression plasmids for the overexpression of a large number of key genes in the early stage of modifications in engineered strains, the optimization of promoters will be adopted as the next strategy to enhance the expression levels of functional genes for further improving the productivity of target metabolites.

In order to improve cell permeability, various genes related to the transmembrane transport were tested by genetic modifications. The most effective modification was the deficiency of MmpL3. We confirmed that the assembly of mycolic acids of the cell envelope in the modified strain was partly inhibited due to the deletion of *mmpL3* (Additional file 2: Figure S3). Thus, the MmpL3 was probable not the sole transmembrane transporter for TMM in *M. neoaurum*. If it was the sole transmembrane transporter for TMM, the growth of the *mmpL3*-deficient strain would display a drastic decline due to the complete inhibition of TMM donation [14]. In addition, the deletion of *mmpL3* was proved to be a useful strategy to interfere in the normal cell envelope assembly. It is important to test whether the interference of the biosynthesis way of intracellular mycolic acids and the subsequent re-assembly of TMM, mediated by the antigen 85 proteins (FbpABC) [36] after TMM molecules were transported into the periplasm, would be beneficial to the improvement in cell permeability.

## Conclusions

The deletion of *mmpL3* did elevate cell permeability without leading to an obvious growth inhibition. Both the utilization of sterol substrates and the production of 4-HBC in corresponding strains were significantly improved. Moreover, the combined modifications of CYP125, ChoM1, and FadA5, in the upstream pathway of sterol to 4-HBC, further enhanced the 4-HBC productivity.

## Methods

### Strains, plasmids, and culture conditions

The strains and plasmids used in this study are described in Table 1. The primers used for construction of the modified strains are described in Additional file 1: Table S2. *Escherichia coli* DH5α was used for plasmid replication. All *M. neoaurum* strains used here were derived from ATCC 25795. A typical 4-HBC-producing strain WIII (Δ*kshA*Δ*hsd4A*Δ*kstD123*) was constructed by deleting *kshAs*, *hsd4A*, *kstD1*, *kstD2*, and *kstD3* based on unmarked allelic homologous recombination [8]. The strain Δ*mmpL3* was constructed by deleting *mmpL3* in *M. neoaurum* ATCC 25795. The engineered strain WIIIΔ*mmpL3* was constructed by deleting *mmpL3* in the WIII strain based on the allelic recombination.

**Table 1:** Strains and plasmids used in this study

Name	Description	Source
Strains		
*M. neoaurum* ATCC 25795	Type strain of *M. neoaurum*	ATCC
Δ*mmpL3*	*mmpL3* deleted in *M. neoaurum* ATCC 25795	This study
Δ*mmpL3*+*mmpL3*	*mmpL3* complemented in strain Δ*mmpL3*	This study
WIII	*kshAs*, *hsd4A*, *kstD1, kstD2* and *kstD3* deleted in *M. neoaurum* ATCC 25795	[30]
WIIIΔ*mmpL3*	*kshAs*, *hsd4A*, *kstD1, kstD2, kstD3* and *mmpL3* deleted in *M. neoaurum* ATCC 25795	This study
WIIIΔ*mmpL3*-*cyp125* -*choM1*-*fadA5*	*cyp125*, *choM1*and *fadA5* overexpressed in strain WIIIΔ*mmpL3*	This study
Plasmids		
p2NIL	Vector of two homologous arms for allelic recombination in mycobacteria, *Kan* ^*R*^	[37]
p2N-*mmpL3*	p2NIL carrying two homologous arms of *mmpL3*, *Kan* ^*R*^	This study
pGOAL19	*Hyg,* Pag85-*lacZ,* P_*hsp60*_-*sac*B, *Pac*I cassette vector, *Amp* ^*R*^	[37]
p19-*mmpL3*	p2NIL-derived with selection cassette from pGOAL19 for deletion of *mmpL3* in mycobacteria	This study
pMV261	Shuttle vector of *Mycobacterium* and *E. coli,* carrying the heat shock (*hsp60*) promoter, *Kan* ^*R*^	Dr. W. R. Jacobs Jr.
p261-*choM1*	Recombinant pMV261, for overexpression of ChoM1 in mycobacteria	This study
p261-*choM2*	Recombinant pMV261, for overexpression of ChoM2 in mycobacteria	This study
p261-*cyp125*	Recombinant pMV261, for overexpression of CYP125 in mycobacteria	This study
p261-*fadA5*	Recombinant pMV261, for overexpression of FadA5 in mycobacteria	This study
p261-*cyp125*-*choM1*-*fadA5*	Recombinant pMV261, for overexpression of CYP125, ChoM1, and FadA5 in mycobacteria	This study
pMV306	Integration vector in *Mycobacterium*, without promoter, *Kan* ^*R*^	Dr. W. R. Jacobs Jr.
p306-*mmpL3*	pMV306-P*hsp60*-*mmpL3*, integrative into mycobacterial chromosomal DNA	This study

*Escherichia coli* DH5α was cultured at 37 °C in 5 mL of Luria–Bertani (LB) medium (10.0 g/L tryptone, 5.0 g/L yeast extracts, 10 g/L NaCl, pH 7.0). Mycobacteria cells were firstly grew in 5 mL of LB medium (OD_600__nm_ = 1.0–1.8). Then, cell suspensions were inoculated into (inoculum volume 1:10, v/v) 30 mL of MYC/01 medium (20.0 g/L glycerol, 2.0 g/L citric acid, 0.05 g/L ammonium ferric citrate, 0.5 g/L K_2_HPO_4_, 0.5 g/L MgSO_4_·7H_2_O, 2.0 g/L NH_4_NO_3_, pH7.5) in 250-mL flasks to prepare the mycobacterial strains (OD_600 nm_ = 1.2–1.8).

For phenotypic identification, the cultivated mycobacterial strains were inoculated to (inoculum volume 1:10, v/v) 30 mL of MM (minimal medium, ammonium ferric citrate 0.05 g/L, K_2_HPO_4_ 0.5 g/L, MgSO_4_·7H_2_O 0.5 g/L, NH_4_NO_3_ 2.0 g/L) with 1 g/L of cholesterol (purity >95.0%, Aladdin Reagents (Shanghai) Co., Ltd., Shanghai, China). For vegetative cell biotransformation, the cultivated mycobacterial strains were transferred into (inoculum volume 1:10, v/v) 30 mL of MYC/02 medium (10.0 g/L glucose, 2.0 g/L citric acid, 0.05 g/L ferric ammonium citrate, 0.5 g/L MgSO_4_·7H_2_O, 2.0 g/L NH_4_NO_3_, pH7.5) with 2 g/L of phytosterols (purity >95.0%, 100 g phytosterol contains 47.5 g β-sitosterol, 26.4 g campesterol, 17.7 g stigmasterol, 3.6 g brassicasterol and 4.8 g undetermined components) (Zhejiang Davi Pharmaceutical Co., Ltd., Zhejiang, China). Before steroid conversion, cholesterol or phytosterol (100.0 g/L) was firstly emulsified in Tween 80 (5% w/v) aqueous solution at 121 °C for 60 min and then added into MM or MYC/02 medium. All of the shake flask experiments were carried out at 30 °C with a shaking speed of 200 rpm in aerobic conditions for 120 h. For resting cell transformation, the cultivated mycobacterial strains were transferred into (inoculum volume 1:10, v/v) 150 mL of MYC/02 medium in 1000-mL shake flasks. After three days of growth at 30 °C under a shaking speed of 200 rpm, the cells were harvested by centrifugation at 8000×*g* for 15 min, washed with 20 mM KH_2_PO_4_, and diluted into cell suspensions (200 g/L). Resting cell transformation was performed in the system containing 100 g/L mycobacterial cells, 20 g/L phytosterols, and 80 g/L hydroxypropyl-β-cyclodextrin (HP-β-CD, RSC Chemical Industries Co. Ltd., Jiangsu, China) under non-sterile conditions in 250-mL flasks at 30 °C and 200 rpm [31]. Standard reference 4-HBC was purified and identified by ourselves [8]. Other solutions and reagents were prepared according to the previously described method [24, 30].

### Gene deletion, overexpression, and complementation in *M. neoaurum*

Target gene-deleted mutant strains were obtained via unmarked homologous recombination strategy in mycobacteria as previously described [23]. Plasmids of p2 N-*mmpL3* and p19-*mmpL3* were used for the knockout of *mmpL3* (Table 1) [37].

To generate the target gene-overexpressed strains, the p261-gene was constructed as previously described [23]. The recombination of p261-genes was transferred into the WIIIΔ*mmpL3*, respectively (Table 1). After PCR analysis using primer pair O-p261-F and O-p261-R, the correct monoclonal strains with sole gene-overexpressed were confirmed.

To complement the expression of target genes, the functional complementation recombinant based on pMV306 was constructed and complemented in the deletion strain according the previous method [23]. The expression cassette of *mmpL3,* containing a heat shock promoter *hsp60*, from the wild-type *M. neoaurum* (WT) was integrated into the double digestion sites of pMV306. Subsequently, this constructed plasmid p306-*mmpL3* was transferred into the *mmpL3*-deleted mutant strain to complement the MmpL3 function.

### Permeability analysis of the cell envelope

The permeability of the cell envelope was examined by measuring the fluorescence intensity of the cells labeled by fluorescein diacetate (FDA, Aladdin Reagents (Shanghai) Co., Ltd., Shanghai, China) according to previous procedures [38]. Cell suspensions (cell density reached 10^6^ cells/mL) of 4.0 mL was mixed with 0.5 mL FDA acetone solution (2 mg/mL) and vibrated at 32 °C for 5 min before detection with a Fluoroskan Ascent Fluorescence Spectrophotometer (Thermo Labsystems Inc., PA, USA). The maximal excitation wavelength for FDA was 485 nm and the emission wavelength was 538 nm.

### Analytical procedures of the mycolic acid methyl esters (MAMEs)

The MAMEs from the *M. neoaurum* cells were obtained according to previous procedures [36]. To avoid the possible interference from polar lipids, the cells (50 mg, in wet weight) were collected at 12,000×*g* for 10 min and then 0.5 mL of the mixture of methanol and chloroform (2:1, v/v) was added. The homogeneous single-phase mixture was incubated for 2 h at 60 °C and then the mixture containing the delipidated cells was centrifuged at 8000×*g* for 10 min. The MAMEs were prepared from the delipidated cells by incubation in 500 μL of 10% tetrabutylammonium hydroxide (Sigma-Aldrich LLC., MO, USA) overnight at 100 °C. After cooling, the mixtures were diluted with 500 μL of water, 250 μL of dichloromethane, and 62.5 μL of iodomethane (Sigma-Aldrich LLC., MO, USA), stirred for 30 min, and then centrifuged at 12,000×*g* for 10 min. The upper layer was removed and the lower organic layer was then washed with 1.0 mL of hydrochloric acid (1 M), followed by 1.0 mL of water. Subsequently, the reaction solution was dried under a stream of nitrogen to obtain crude MAMEs. The residue was dissolved in a mixture of toluene (0.2 mL) and acetonitrile (0.1 mL), followed by the addition of acetonitrile (0.2 mL), and then incubated for 1 h at 4 °C. The MAMEs were obtained by centrifugation and then re-suspended in dichloromethane.

The MAMEs were then analyzed by TLC on aluminum-backed silica gel 60-precoated plates F254 (Merck & Co., Inc., Hesse-Darmstadt, Germany) in a solvent system (chloroform: methanol, 90:10, v/v). The spots on the plates were observed after heating with cupric sulfate (10% w/v in an 8% v/v phosphoric acid solution).

### Sterol transformation, sample extraction, and analysis

In this work, two methods including vegetative cell biotransformation and resting cell transformation were used for the assessment of steroid conversion capability [23, 31]. The conversion system was sampled (0.5 mL from the vegetative cell biotransformation; 0.1 mL from the resting cell transformation) every 24 h. The samples from the vegetative cell biotransformation were extracted with 0.5 mL of ethyl acetate and the samples from the resting cell transformation were extracted with 1.0 mL of ethyl acetate.

For gas chromatography (GC) analysis, a GC system 7820A (Agilent Technologies, CA, USA) was used in the quantitative determination of cholesterol and the mixture of phytosterols. The ethyl acetate extracts (5 μL) from the samples were injected into a DB-5 column (30 m × 0.25 μm (i.d.) × 0.25 μm film thickness, Agilent Technologies, CA, USA). The oven temperature was programmed as follows: 200 °C for 2 min, 200–280 °C within 4 min, 280 °C for 2 min, 280–305 °C within 1.5 min, and 305 °C for 10 min. Inlet and flame-ionization detector temperatures were maintained at 320 °C. Nitrogen carrier gas flow was 2 mL/min at 50 °C.

For high performance liquid chromatography (HPLC) analysis, the extracts of the samples containing 4-HBC were transferred into clean tubes, dried under vacuum, re-dissolved in methanol, and then centrifuged at 12,000×*g* for 20 min. The prepared samples were analyzed with a reversed-phase C18-column (250 × 4.6 mm) (Agilent Technologies, CA, USA) at 254 nm with an Agilent 1100 series HPLC (Agilent Technologies, CA, USA). The mixture of methanol and water (80:20, v/v) was used as the mobile phase.

## Additional files

**Additional file 1: Table S1.** Comparison of *mmpL3* region of mycobacteria. **Table S2.** Primers used in this study.

**Additional file 2: Figure S1.** Effects of *mmpL3* deficiency on the uptake of steroids in *M. neoaurum* ATCC 25795. **Figure S2.** Effects of the deletion of *mmpL3* on 4-HBC-producing strains. **Figure S3.** Effects of *mmpL3* deficiency on the content of mycolic acids in the cell envelope.

## Abbreviations

4-HBC: 22-hydroxy-23,24-bisnorchol-4-ene-3-one
TMM: trehalose monomycolate
ChoM: cholesterol oxidase
TDM: trehalose dimycolate
MAMEs: mycolic acid methyl esters
CYP125: cytochrome P450 125
FadA5: thiolase FadA5
1,4-HBC: 22-hydroxy-23,24-bisnorchol-1,4-dien-3-one
9-OHHBC: 9,22-dihydroxy-23,24-bisnorchol-4-ene-3-one
AD: androst-4-ene-3,17-dione
ADD: androst-1,4-diene-3,17-dione
9-OHAD: 9α-hydroxy-4-androstene-3,17-dione
LB: Luria–Bertani
MM: minimal medium
FDA: fluorescein diacetate
HPLC: high performance liquid chromatography
GC: gas chromatography
