# Supplementary material for: Improving the production of 22-hydroxy-23,24-bisnorchol-4-ene-3-one from sterols in Mycobacterium neoaurum by increasing cell permeability and modifying multiple genes
Source: Microb Cell Fact. 2017 May 22;16:89. doi: 10.1186/s12934-017-0705-x (PMC5440992; doi:10.1186/s12934-017-0705-x)
Supplement: Supplementary file 1 — Additional file 1: Table S1. Comparison of mmpL3 region of mycobacteria. Table S2. Primers used in this study. [file 12934_2017_705_MOESM1_ESM.docx]

**Additional file 1**

Improving the production of 22-hydroxy-23,24-bisnorchol-4-ene-3-one from sterols in *Mycobacterium neoaurum* by increasing cell permeability and modifying multiple genes

Liang-Bin Xiong^1^, Hao-Hao Liu^1^, Li-Qin Xu, Wan-Ju Sun, Feng-Qing Wang^*^ and Dong-Zhi Wei^*^

State Key Laboratory of Bioreactor Engineering, Newworld Institute of Biotechnology, East China University of Science and Technology, Shanghai 200237, China

Running Head: Increase 4-HBC productivity in *M. neoaurum*

^1^ These authors contributed equally to this work.

^*^ Address correspondence to Feng-Qing Wang, fqwang@ecust.edu.cn; Dong-Zhi Wei, dzhwei@ecust.edu.cn

Email addresses for other authors:

Liang-Bin Xiong: lbxiong2010@163.com

Hao-Hao Liu: hhliu2012@163.com

Li-Qin Xu: liqin_xu@yeah.net

Wan-Ju Sun: sunwj4135@126.com

**Table S1** Comparison of *mmpL3* region of mycobacteria

| *M. neoaurum* ATCC 25795 | *M. neoaurum* NRRL B-3805 (ID) | *M. neoaurum* VKM Ac-1815D (ID) | *M. tuberculosis* H37Rv (ID) | Annotation |
| --- | --- | --- | --- | --- |
| *Mn_1719* | *MyAD_02710* (93%) | *D174_02775*  (93%) | *Rv0204c*  (70%) | Transmembrane protein |
| *Mn_1720* | *MyAD_02715* (92%) | *D174_02780*  (92%) | *Rv0205*  (72%) | Transmembrane protein |
| *mmpL3* | *MyAD_02720* (94%) | *D174_02785*  (94%) | *mmpL3*  (72%) | Transmembrane transport protein MmpL3 |
| *Mn_1722* | *MyAD_02725*  (92%) | *D174_02790*  (92%) | *Rv0207c*  (80%) | Hypothetical protein |
| *Mn_1723* | *MyAD_02730*  (92%) | *D174_02795*  (92%) | *Rv0208c*  (71%) | tRNA (guanine(46)-N(7))-methyltransferase |

**Table S2** Primers used in this study

| **Primers** | **Description** |
| --- | --- |
| **For gene deletion** |  |
| D-*mmpL3*-UF | TATAaagcttGCCTTGATCGCCGACCGTCTCCGAC |
| D-*mmpL3*-UR | GCGCgaattcGACACCGATGACTATGTATCGGTAC |
| D-*mmpL3*-DF | TATAgaattcCCTGAGCCGACGGAGAAGCTCGACA |
| D-*mmpL3*-DR | TATAggatccCGACGATGTTCGCAGCACTGCTGAC |
| **For** **gene complementation** |  |
| C-*mmpL3*-F | TATActgcagAAGTGTTCGCCTGGTGGGGTCGA |
| C-*mmpL3*-R | GATAaagcttTTACAGACGGCCCTCCCGGCGAA |
| **For gene overexpression** |  |
| O-p261-F | TAGGCGAGTGCTAAGAATAACGTT |
| O-p261-R | TCGTTTTATTTGATGCCTGGCAGT |
| O-*choM1*-F | GTAgaattcATGAAGCCTGACTATGACGT |
| O-*choM1*-R | TATgtcgacCTAAGCCGAAGGTGACAGCT |
| O-*choM2*-F | CGAgaattcTTGCTGACAAGACGGCGGTT |
| O-*choM2*-R | TATgtcgacCTACTTGCGGCCCTGCAGGA |
| O-*cyp125*-F | TCAgaattcGTGGTACTAGAAGGAATTGT |
| O-*cyp125*-R | TATgttaacCTAGCTCGAGGCGCCGGCGC |
| O-*fadA5*-F | TATggatccAATGGGTAATCCTGTCATCG |
| O-*fadA5*-R | TGTgaattcTTAGATCCGCTCGATGATGG |
| O-*choM1*-SD-F | TATaagcttaagaaggagatataATGAAGCCTGACTATGACGT |
| O-*choM1*-R | TATgttaacCTAAGCCGAAGGTGACAGCT |
| O-*fadA5*-SD-F | TCGgttaacaagaaggagatataATGGGTAATCCTGTCATCGT |
| O-*fadA5*-R | TCGgttaacTTAGATCCGCTCGATGATGG |
