# Supplementary material for: Improving the production of 22-hydroxy-23,24-bisnorchol-4-ene-3-one from sterols in Mycobacterium neoaurum by increasing cell permeability and modifying multiple genes
Source: Microb Cell Fact. 2017 May 22;16:89. doi: 10.1186/s12934-017-0705-x (PMC5440992; doi:10.1186/s12934-017-0705-x)
Supplement: Supplementary file 2 — Additional file 2: Figure S1. Effects of mmpL3 deficiency on the uptake of steroids in M. neoaurum ATCC 25795. Figure S2. Effects of the deletion of mmpL3 on 4-HBC-producing strains. Figure S3. Effects of mmpL3 deficiency on the content of mycolic acids in the cell envelope. [file 12934_2017_705_MOESM2_ESM.docx]

**Additional file 2**

Improving the production of 22-hydroxy-23,24-bisnorchol-4-ene-3-one from sterols in *Mycobacterium neoaurum* by increasing cell permeability and modifying multiple genes

Liang-Bin Xiong^1^, Hao-Hao Liu^1^, Li-Qin Xu, Wan-Ju Sun, Feng-Qing Wang^*^ and Dong-Zhi Wei^*^

State Key Laboratory of Bioreactor Engineering, Newworld Institute of Biotechnology, East China University of Science and Technology, Shanghai 200237, China

Running Head: Increase 4-HBC productivity in *M. neoaurum*

^1^ These authors contributed equally to this work.

^*^ Address correspondence to Feng-Qing Wang, fqwang@ecust.edu.cn; Dong-Zhi Wei, dzhwei@ecust.edu.cn

Email addresses for other authors:

Liang-Bin Xiong: lbxiong2010@163.com

Hao-Hao Liu: hhliu2012@163.com

Li-Qin Xu: liqin_xu@yeah.net

Wan-Ju Sun: sunwj4135@126.com

**Additional Methods**

**Steroid uptake analysis of mycobacterial cells**

The uptake of steroids in the *mmpL3-*deficient *M. neoaurum* ATCC 25795 was determined by measuring the amount of cholest-4-en-3-one entering the cells per unit of time. Before use, cholest-4-en-3-one was emulsified in Tween 80 (5% w/v) aqueous solution at 121 °C for 60 min. Cultivation conditions were the same as described in the paper. The cultivation system containing 1.0 g/L cholest-4-en-3-one was sampled after 12 h of growth (at least 5 mL). Samples were centrifuged at 12000×g for 10 min, washed with 1.0 mL of ddH_2_O for two times, and washed with 1.0 mL of the mixture of petroleum ether and ethyl acetate (60:40, v/v) to remove the cholest-4-en-3-one in culture media. Then, the cells (50 mg, in wet weight) were suspended with 1.0 mL of acetonitrile and ddH_2_O (70:30, v/v). After adding 0.8 g of glass beads, the cells were broke with FastPrep-24 Instrument (MP Biomedicals, CA, USA) and centrifuged at 12000×g for 10 min. Cholest-4-en-3-one entering cells could be released and dissolved in the acetonitrile solution. The extracts were analyzed by thin-layer chromatography (TLC) on aluminum-backed silica gel 60-precoated plates F254 (Merck & Co., Inc., Hesse-Darmstadt, Germany) in a solvent system (petroleum ether: ethyl acetate, 80:20, v/v). The spots on the plates were observed under UV light. Besides, the extracts (30 μL) were analyzed with a reversed-phase C18-column (250 mm × 4.6 mm) at 254 nm with an Agilent 1100 series HPLC. The methanol was used as the mobile phase.

**Figure S1**

**
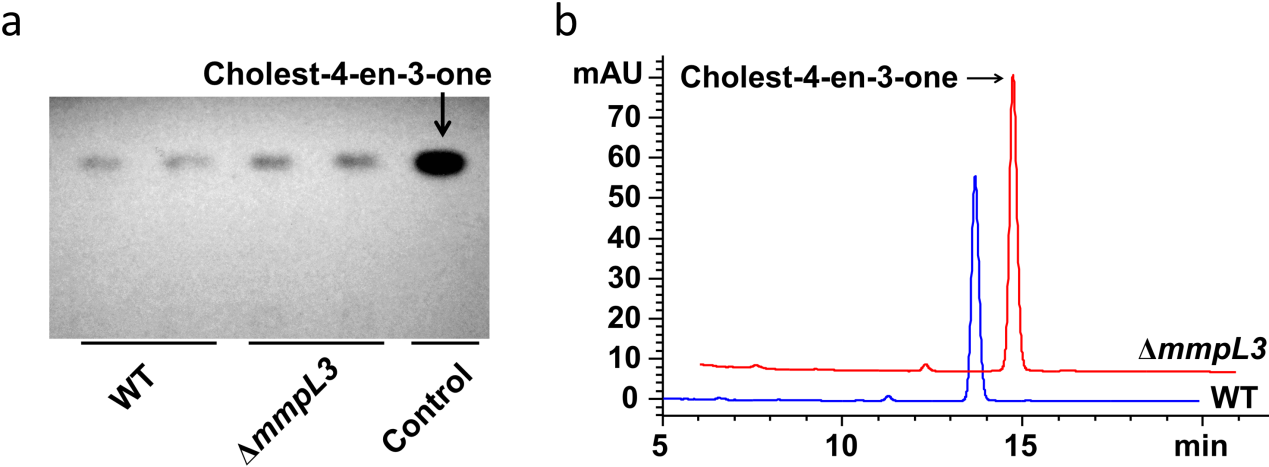
**

**Figure S1** Effects of *mmpL3* deficiency on the uptake of steroids in *M. neoaurum* ATCC 25795. The cholest-4-en-3-one entered into the mycobacterial cells after 12 h of growth in MM containing 1.0 g/L cholest-4-en-3-one was assessed. **a**. TLC analyses of cholest-4-en-3-one in the wild-type strain (WT) and the *mmpL3-*deficient strain (Δ*mmpL3*). **b**. HPLC analyses of cholest-4-en-3-one in the two strains. The uptake of cholest-4-en-3-one was increased about 33.7%.

**Figure S2**

**

**

**Figure S2** Effects of the deletion of *mmpL3* on the phytosterol utilization in the 4-HBC-producing strain. Quantitative analyses of the residual phytosterols, when the strains were cultured in MYC/02 medium with 2.0 g/L phytosterols addition. Data represent the mean ± standard deviation of three measurements.

**Figure S3**

**
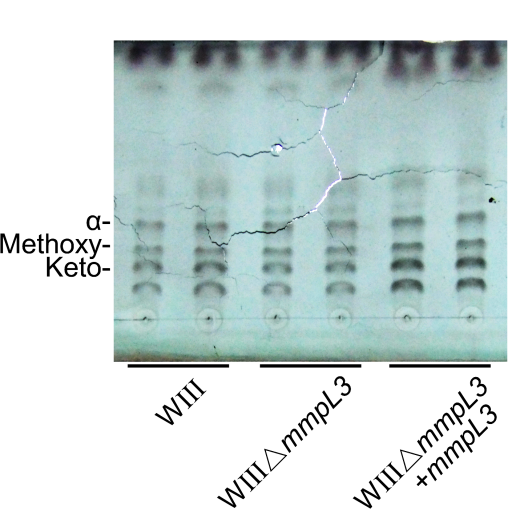
**

**Figure S3** Effect of *mmpL3* deficiency on the content of mycolic acids in the cell envelope. The typical 4-HBC-producing strain WIII, the *mmpL3* deleted strain WIIIΔ*mmpL3* and the *mmpL3* complemented strain WIIIΔ*mmpL3*+*mmpL3* were cultured in MYC/02 medium with 1.0 g/L phytosterols. The MAMEs (α-, methoxy- and keto- are three forms of mycolic acids in *M. neoaurum*) was isolated from the cells after the extractable lipids were removed. The same amount of the extracts was used similarly and the plates were revealed with cupric sulfate (10% w/v in an 8% v/v phosphoric acid solution).
